# Detection of Cancer‐Associated Mutations Using Primer Exchange Reaction‐Based Signal Amplification and Lateral Flow Assays

**DOI:** 10.1002/smsc.202500520

**Published:** 2026-02-06

**Authors:** Samet Kocabey, Curzio Rüegg

**Affiliations:** ^1^ Laboratory of Experimental and Translational Oncology Department of Oncology Microbiology and Immunology Faculty of Science and Medicine University of Fribourg Fribourg Switzerland; ^2^ NCCR Bio‐inspired Materials University of Fribourg Fribourg Switzerland; ^3^ Xemperia SA, Marly Innovation Centre Marly Switzerland

**Keywords:** circulating tumor DNA, DNA self‐assembly, lateral flow assay, liquid biopsy, mutation detection, primer exchange reaction, RNA, signal amplification

## Abstract

The ability to sensitively and specifically detect cancer‐associated nucleic acids carrying single‐nucleotide mutations is critical for early cancer detection, patient stratification, and personalized treatment, particularly through non‐invasive liquid biopsy approaches. Detecting low‐abundance nucleic acid fragments—particularly those with single‐nucleotide variations—remains a significant challenge for point‐of‐care (POC) diagnostics. Here, we report a programmable DNA‐based self‐assembly strategy that leverages primer exchange reaction (PER) for isothermal signal amplification and enables colorimetric detection of cancer‐specific DNA and RNA fragments on gold nanoparticle‐based lateral flow assays (LFAs). This method uses PER‐generated DNA concatemers functionalized with multiple FITC‐labeled imager strands to enhance the visual signal on conventional LFA strips. We demonstrate that this approach enables detection of a synthetic *P53* oncogene fragment with a limit of detection as low as 16 pM, representing a 16‐fold improvement over single‐dye labeled controls. The system also reliably distinguishes single‐nucleotide mutations at 10% relative abundance within a wild‐type background. Moreover, we show successful detection of mutant fragments in complex biological fluids such as serum and saliva, as well as of RNA extracted from breast cancer cell lines and RNA derived from circulating tumor DNA (ctDNA) from patient plasma samples. Specifically, we detect clinically relevant *PIK3CA* E545K/A and *P53* R280K mutations, consistent with Sanger sequencing results and validating our method for liquid biopsy applications. Overall, this PER‐based self‐assembly system provides a simple, robust, and sensitive platform for mutation‐specific nucleic acid detection using LFAs and offers strong potential for translation into laboratory research applications and POC diagnostics workflows for cancer and other genetic disorders.

## Graphical abstract

This article presents the detection of cancer‐specific mutations using primer exchange reaction‐based signal amplification on lateral flow assays. This approach enables sensitive detection of clinically relevant mutations, including PIK3CA E545K/A and TP53 R280K, from RNA isolated from breast cancer cell lines and from RNA derived from circulating tumor DNA (ctDNA) in patient plasma samples.

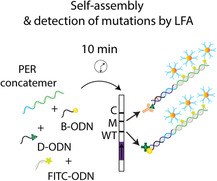

## 1 Introduction

Single DNA mutations, known as point mutations, can alter the amino acid sequence by substituting one nucleotide for another or by inserting or deleting a single nucleotide [1]. These mutations can significantly affect the resulting protein's structure and function, potentially disrupting downstream proteins and leading to various diseases, including cancer [2]. Early and accurate detection of cancer‐associated genetic mutations is critical for early cancer detection, patient's stratification, treatment, and monitoring of minimal residual disease [3, 4, 5]. Among various biomolecular targets, nucleic acids carrying cancer driver mutations serve as highly specific biomarkers for malignancies [6, 7, 8]. Circulating tumor DNA (ctDNA), released into bodily fluids by cancer cells, represents an easily accessible biomarker that can provide dynamic insights into tumor burden and molecular evolution [9]. However, ctDNAs are often present at very low concentrations in the bloodstream and may differ from wild‐type sequences by only a single nucleotide, posing significant analytical challenges for detection, especially in resource‐limited laboratories or in point‐of‐care (POC) settings [10, 11].

While traditional molecular diagnostic techniques such as quantitative polymerase chain reaction (PCR) (qPCR), digital PCR and next‐generation sequencing offer high sensitivity and specificity, their reliance on sophisticated instrumentation, trained personnel, and extended processing times limit their accessibility in clinical routine as well as laboratory research [12, 13, 14]. Lateral flow assays (LFAs), on the contrary, are inexpensive, easy to operate, and enable rapid visual readouts without the need for instrumentation suitable for decentralized diagnostics [15, 16, 17, 18]. However, conventional LFAs often exhibit limited sensitivity (micromolar range) and specificity compared to standard laboratory methods such as enzyme‐linked immunosorbent assays (picomolar to femtomolar) and PCR (attomolar to yoctomolar) [16, 19]. Additionally, their poor adaptability for nucleic acid detection—due to the need for extensive sample preparation or signal amplification strategies—further limits their clinical effectiveness.

To overcome these limitations, there is growing interest in integrating isothermal amplification and DNA nanotechnology into LFA platforms to improve analytical performance. Very recently, a variety of signal amplification strategies have been developed to enhance the sensitivity of biomarker detection on LFAs including the use of hybridization chain reaction, rolling circle amplification (RCA), recombinase polymerase amplification (RPA), loop‐mediated isothermal amplification (LAMP), branched DNA polymers, and DNA origami structures [20, 21, 22, 23, 24, 25, 26]. However, many of these strategies mainly rely on an on‐going reaction, they are incompatible with a ready‐to‐use design with higher controllability, they can require complex primer design (e.g. LAMP), circular DNA template (e.g. RCA) or more expensive reagents (e.g. RPA) [27]. Moreover, the in situ enzymatic reaction could be hard to control or tune for individual targets. By contrast, primer exchange reaction (PER) stands out as a powerful DNA‐based signal amplification method that operates under mild, isothermal conditions and enables programmmable, modular synthesis of single‐stranded DNA concatemers [28, 29]. These concatemers can be functionalized with reporter sequences to enhance detection signals on LFAs without requiring enzymes or thermal cycling at the point of detection.

In this study, we report the development of a PER‐driven signal amplification and self‐assembly approach for the colorimetric detection of cancer‐associated nucleic acid targets using commercial gold nanoparticle‐based LFA strips. By leveraging the programmable nature of PER‐generated concatemers and their capacity to recruit multiple dye‐labeled imager strands, we enable robust signal amplification that results in a visible readout within minutes for detecting single‐stranded DNA and RNA fragments carrying oncogenic mutations. Combining PER‐driven self‐assembly with conventional LFAs enables the detection of low picomolar concentrations of target sequences, single‐base discrimination, and use in complex biological matrices such as serum and saliva. Remarkably, the approach allows detection of mutations in plasma‐derived ctDNA from breast cancer patients. The simplicity of the workflow, which is compatible with room temperature operation and standard lateral flow materials, highlights the potential of this method for translation into practical research and diagnostic tools for resource‐limited environments. In the following sections, we describe the development, optimization, and validation of this approach, including its ability to detect clinically relevant mutations in P53 and PIK3CA genes, both from synthetic oligonucleotides (ODNs) and real biological samples. We further demonstrate its capacity to discriminate mutant alleles within wild‐type backgrounds, supporting its potential for integration into POC diagnostics and liquid biopsy workflows.

## 2 Results and Discussion

### 2.1 PER‐Based Target Detection Principle on LFAs

To achieve rapid and sensitive detection of cancer‐associated nucleic acids carrying cancer driver mutations on LFAs, we developed a DNA‐based self‐assembly approach using a programmable PER‐based signal amplification that allows colorimetric detection. For this, we used conventional lateral flow strips comprising a nitrocellulose membrane striped with capture reagents ‐streptavidin and anti‐digoxigenin antibody for capturing target labeled ODNs and polyclonal anti‐goat antibody for capturing control anti‐FITC‐Ab‐AuNP‐ as well as sample application pad (SAP), which contains the gold nanoparticles responsible for the visualization of the test and control line. The gold nanoparticles on the SAP have a diameter of 40 nm and are coupled with polyclonal goat anti‐FITC antibodies.

**FIGURE 1:**
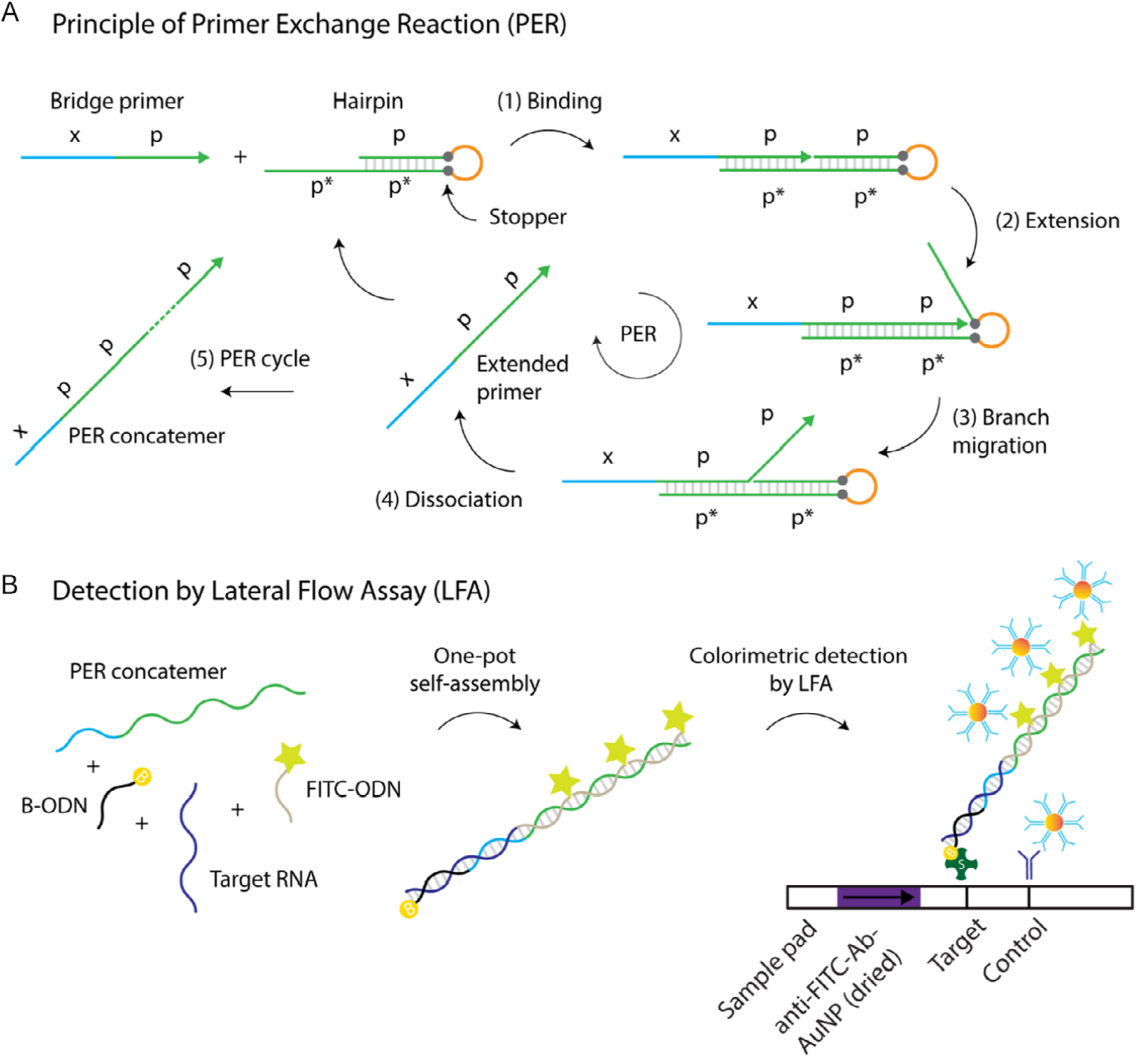
Schematic illustration of the (A) PER principle and (B) the detection of target RNAs on LFAs using PER‐based signal amplification.

To capture target ODN on the lateral flow strips, the biotinylated ODN is designed in a way that the target ODN can bind partially to the biotinylated ODN. The remaining single‐stranded unhybridized part of the target ODN is designed to form a duplex with the x domain of the bridge primer (xp) which is used for elongation through PER (Figure 1A). To produce the PER concatemer, which recognizes the target ODN, the bridge primer first binds (1) to the p* domain of the respective hairpin ODN and extended (2) isothermally until encountering with the stopper base‐pair in the presence of strand‐displacing DNA polymerase (e.g. Bst polymerase). The stopper is made of a G–C pair, and the on‐going polymerization stops at this point due to lack of dGTP in the solution, which induces the dissociation of the newly synthesized sequence (xpp) from the hairpin through the branch migration (3). This leads to spontaneous separation between the extended primer and hairpin (4) and the extended primer (xpp) rejoins a next cycle for further elongation after this separation (5). Finally, after multiple PER cycle, a long single‐stranded DNA concatemer with repeated p domains formed, which induce signal amplification through hybridization of FITC‐labeled imager strands.

For running lateral flow assay, presynthesized PER concatemers (Figure 1A) are mixed with biotinylated ODNs, target ODNs, and FITC‐labeled imager strands by one‐pot self‐assembly reaction at room temperature. This solution containing preassembled ODNs is then mixed with running buffer, and lateral flow strips are dipped into this solution through the sample pad region to visualize the detection of target ODNs. The dipping of the strips induces a capillary flow that moves all the components to the test and control lines, where they can interact with the capture reagents. A colorimetric signal, which is visible to the naked eye, is generated when many anti‐FITC‐Ab labeled gold nanoparticles bind to the FITC containing amplification domains of the PER concatemer (Figure 1B).

### 2.2 Determining the Detection Sensitivity Using PER

**FIGURE 2:**
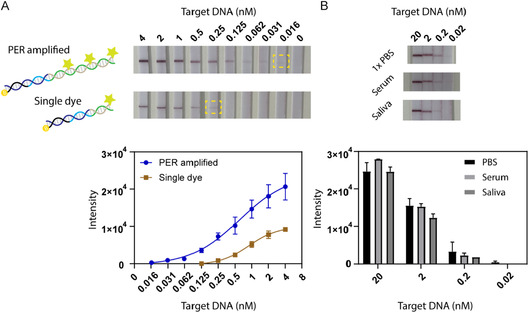
Detection sensitivity on lateral flow strips. (A) Detection of *P53* DNA oligonucleotide on lateral flow strips by PER‐based signal amplification compared to single‐dye labeling. Visual LoDs are highlighted. (B) Detection of *P53* DNA oligonucleotide in various biological fluids. In the bottom panels, test line intensities are plotted against the target DNA concentration. The error bars represent the standard deviation obtained from three independent measurements.

Next, we investigated the detection sensitivity of our DNA self‐assembly approach using PER‐based signal amplification. As a proof‐of‐concept experiment, we used a synthetic single‐stranded DNA fragment from the human *P53* proto‐oncogene and employed a linear signal amplification strategy for the detection. This was compared to detection using a single FITC‐labeled ODN. For this, we applied final target ODN concentrations ranging from 4 nM to 16 pM (Figure 2A). The target sequences hybridized to the biotinylated ODNs and PER‐generated concatemers, which were then captured by streptavidin on the lateral flow strips. As an unamplified control, we used a non‐elongated primer complementary to the single FITC‐labeled ODN, applied at the same concentration as the PER concatemers carrying multiple FITC dyes. The test line images (Figure 2A, top) and the corresponding target line intensity values plotted against the target DNA ODN concentration (Figure 2A, bottom) demonstrate that the test line signal could be visually detected for ODN concentration down to 16 pM when amplified using PER. In contrast, with the single dye‐labeled control, detectable signal was only achieved at 256 pM or higher, indicating a 16‐fold improvement in the limit of detection (LoD) with PER‐based amplification. The theoretical LoD of target ODN by PER amplification was further defined as the mean signal intensity of the blank sample plus three standard deviations (3*σ*) of the lowest nonzero concentration. By fitting all the measured data points (4 nM–16 pM) to a 4‐parameter logistic (4PL) nonlinear regression curve, the LoD of *P53* ODN was determined as 16 pM.

In our previous study, we demonstrated that PER concatemers typically range from 300 to 400 nucleotides in length (Figure S1), enabling hybridization of at least 15–20 dye‐labeled imager strands (20‐nt each) achieving fluorescence signal enhancement up to 75‐fold with flow cytometry‐based detection [30]. This difference in LoD between two different detection approaches was expected since the FITC molecules are spaced ≈7 nm apart and the size of gold nanoparticles used in the lateral flow assay are 40 nm in diameter. This size disparity limits the number of anti‐FITC antibodies that can bind each FITC molecule, reducing the overall intensity of the detection signal. We further attempted to improve sensitivity using 5 and 10 nm FITC‐Ab labeled AuNPs by premixing these nanoparticles with self‐assembled PER concatemers. However, the signal intensity was lower than that achieved with 40 nm gold nanoparticles (Figure S2A). Furthermore, we explored a branching strategy for signal amplification involving independently assembled secondary concatemers hybridized to the primary concatemers to increase the number of binding sites of FITC‐labeled strands, but the colorimetric signal remained similar to that of linear amplification, likely due to the steric hinderance and the saturation of FITC‐Ab‐AuNP complexes that bind to FITC molecules (Figure S2B).

Finally, we demonstrated that the detection of target ODNs through PER‐based signal amplification can be performed in complex biological fluids like serum and saliva. For this, target ODN concentrations in the range from 20 nM to 20 pM were spiked into 1× PBS, serum or saliva, mixed with biotinylated ODNs and PER concatemers, and subsequently captured on lateral flow strips via streptavidin. The results showed that the test line signals were detectable down to 200 pM in both serum and saliva, with signal intensities comparable to those observed in 1× PBS (Figure 2B). These findings validate the feasibility of target ODN detection in complex biological fluids using this approach.

### 2.3 Single‐Nucleotide Mutation Detection on Lateral Flow Strips

**FIGURE 3:**
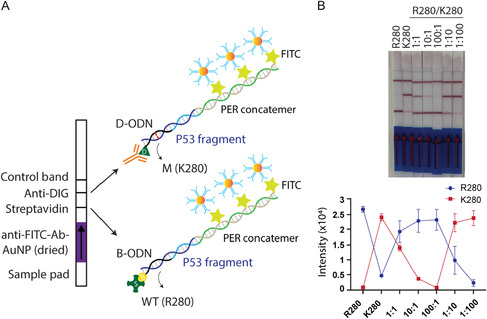
Single‐nucleotide mutation detection on lateral flow strips. (A) Schematic illustration demonstrating the detection of *P53* wild‐type and mutated DNA oligonucleotides on lateral flow strips by PER‐based signal amplification. (B) Detection of *P53* R280 and K280 DNA oligonucleotides in various combinations. In the bottom panel, test line intensities are plotted against the target DNA combinations. The error bars represent the standard deviation obtained from three independent measurements.

After characterization of detection sensitivity of our approach, we applied this technique to assess its detection specificity and its ability to discriminate cancer‐specific mutations. Mutated DNA fragments (e.g. ctDNAs) often coexist with a large background of wild‐type (WT) DNA and they are frequently distinguished by single‐nucleotide variants with allele frequencies as low as 0.01% [11]. To detect these mutations with high precision using PER‐based signal amplification on lateral flow assay, we selected the *P53* R280K mutation for a proof‐of‐concept and designed two synthetic ODNs representing the WT (R280) and mutant (M) (K280) sequences. In order to selective capture of these target ODNs, we used two modified probes: biotinylated ODN for the R280 fragment and digoxigenin‐labeled ODN for the K280 fragment. We then combined these probes with PER‐generated concatemers, FITC‐labeled imager strands, and the target fragments (WT and M *P53*) in various ratios in 1× PBS (WT:M; 1:1, 10:1, 100:1, 1:10, 1:100, only WT and only M) maintaining a constant background concentration of 10 nM for either R280 or K280 (e.g. in 100:1 condition, 10 nM R280 and 100 pM K280). Then, the target sequences hybridized to their respective biotinylated or digoxigenin‐labeled probes and PER‐generated concatemers, which were then captured by streptavidin (lower target line) or anti‐digoxigenin antibody (upper target line) on the lateral flow strips (Figure 3A). The resulting test line images (Figure 3B, top) and the corresponding intensity values for both target lines (WT and M) plotted against the different target ODN (WT and M) combinations (Figure 3B, bottom). The results demonstrate that the test line signal intensity for mutant fragment (K280) could be visually detected even when present at a 10‐fold lower concentration than WT (10:1) with higher signal intensity at the 1:100 ratio. Similarly, test line signal intensity for WT fragment (R280) could also be clearly detected at a 10‐fold lower concentration than the mutant (1:10), again with stronger signal than at 1:100 ratio. Notably, the signal intensities at 1:100 concentrations were comparable to those of the WT only and M only controls. Overall, these results demonstrate that our method can reliably detect mutant fragments at 10% relative abundance, highlighting its potential for detecting mutations within heterozygous DNA populations.

### 2.4 Detection of Single‐Nucleotide Mutations from Cell Extracts and Human Plasma

**FIGURE 4:**
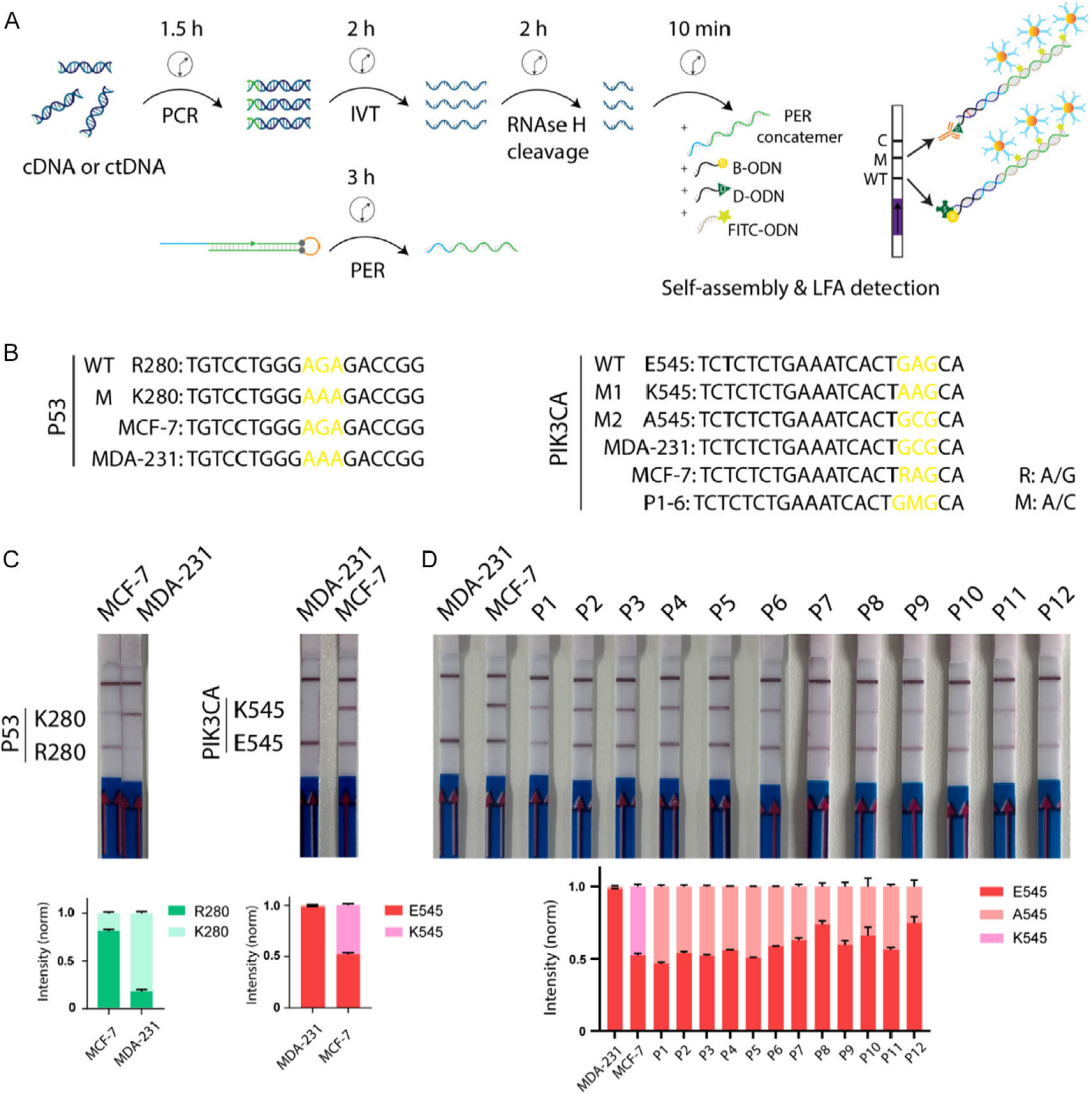
PER‐based detection of breast cancer specific mutations using lateral flow strips. (A) Schematic illustration of the mutation‐detection workflow on lateral flow strips. (B) The list of DNA fragments carrying *P53* and *PIK3CA* gene mutations. (C) Detection of cellular *P53* and *PIK3CA* gene fragments using lateral flow strips. (D) Detection of *PIK3CA* E545A mutations in the plasma of breast cancer patients using lateral flow strips. The bottom panels show normalized test line intensities plotted against the corresponding samples. The error bars represent the standard deviation obtained from three independent measurements.

To assess the specificity of our PER‐based detection approach in biological samples, we next investigated the ability to discriminate wild‐type sequences from single‐nucleotide mutations that occur frequently in cancer. For this, we selected several common mutations frequently seen in breast cancer that we previously screened for mutations by PCR and Sanger sequencing. Among these mutations, missense *PIK3CA* mutations (e.g. E542K, E545K, E545A) are common activating mutations in breast cancer (occurring in 20%–30% of all cases) and are potent predictive markers for responses to PI3K inhibitors in estrogen receptor‐positive, HER2‐negative (ER^+^/HER2^−^) breast cancers [31]. Moreover, *P53* mutants (e.g., R248Q, R273H, and R280K) are classified as contact mutants associated with breast cancer (occurring in 40%–50% of all cases), particularly ER^−^, that are involved in DNA binding without causing protein unfolding but inhibit its transcriptional activity [32]. We first isolated the RNAs from breast cancer cell lines (MDA‐MB‐231 and MCF‐7) and amplified the genes of interest by RT‐PCR (Figure 4A). After sequencing of these amplicons (the sequences are given in Table S2), we observed that *PIK3CA* E545K (heterozygous) and *P53* R280K mutations are present in ER^+^/HER2^−^ and ER^−^/HER2^−^ breast cancer cells, MCF‐7 and MDA‐MB‐231, respectively. In order to detect these mutations using the PER‐based approach on lateral flow assay, we first in vitro transcribed the amplicons and incubated the purified RNA strands with respective guide ODNs for RNase H cleavage (Figure 4A). We used the RNase H cleavage technique instead of directly employing long RNA strands for detection to prevent secondary structure formation in transcribed RNAs, which can mask target sequences and hinder efficient RNA capture [33]. After cleavage, the solution containing fragmented RNA strands with mutations (18 nt for *P53* and 21 nt for *PIK3CA*, as shown in Figure 4B and the detailed in Table S1) was mixed with a solution containing the corresponding biotinylated and digoxigenin‐labeled probes and PER‐generated concatemers. These complexes were then captured on lateral flow strips by streptavidin (lower target line) or anti‐digoxigenin antibodies (upper target line), as illustrated in Figure 4C *.* The results demonstrate the P53 RNA fragments obtained from MDA‐MB‐231 cells predominantly contain the mutant fragment (K280), while those from MCF‐7 cells contain mostly the wild‐type fragment (R280), consistent with the sequencing data (Figure 4C). Similarly, the *PIK3CA* fragments obtained from MDA‐MB‐231 cells predominantly contain the wild‐type fragments (E545), while those from MCF‐7 cells contain both the wild‐type (E545) and mutant (K545) fragments, reflecting the heterozygous state of the *PIK3CA* gene in MCF‐7 cells (Figure 4C). Overall, we demonstrated that that we could efficiently distinguish the aforementioned cancer‐specific mutations using PER‐based signal amplification approach on lateral flow strips.

As we have characterized the detection specificity of our system on cellular extracts, we then employed this technique to identify ctDNAs with target mutations from the plasma of breast cancer patients. Plasma ctDNAs carrying these mutations originated from the cancer cells serve as significant and reliable cancer biomarkers in liquid biopsy, but their concentrations in the bloodstream of cancer patients is extremely low and they are mixed with ctDNA fragments of wild‐type sequences derived from normal cells [10]. For this, we extracted ctDNAs from the plasma of 12 patients with metastatic, ER+ HER2− breast cancer for a proof‐of‐concept experiment. First, we amplified the region of interest of target genes bearing potential mutation (*ESR1:* Y537 and D538*; P53*: R273 and R280; *PIK3CA*: E542 and E545) for all samples (Figure S3) and sequenced the amplicons using Sanger sequencing. After screening of all samples, we only observed a heterozygous *PIK3CA* E545A mutation in the ctDNA extracted from all the patient plasma samples (P1–P12). All the other genes screened were wild‐type. The aligned sequences are given in Figure S4. Then, to distinguish these mutations using PER‐based approach on lateral flow strips, we produced the respective RNA strands from all ctDNA samples (P1–P12) by in vitro transcription (IVT) from the amplified regions and mixed the purified RNA strands with guide ODNs for RNase H cleavage. After cleavage, the solution containing fragmented RNA strands with mutations (Figure 4B) was mixed with a solution containing the corresponding biotinylated and digoxigenin‐labeled probes and PER‐generated concatemers. These complexes were then captured on lateral flow strips by streptavidin (lower target line) or anti‐digoxigenin antibodies (upper target line), as illustrated in Figure 4D. The results showed that the signal intensities of both test lines were comparable, indicating that the heterozygous mutation (E545A) at the corresponding codon (GAG) can be specifically detected in patient samples using PER‐based approach (Figure 4D). In agreement with this observation, Sanger sequencing of the amplicons revealed comparable adenine and cytosine signal intensities corresponding to a heterozygous PIK3CA E545A (A/C) mutation (Figure S4), indicating that our approach did not corrupt the allelic mutant/wild‐type ratio present in the sample. As a negative control, the *PIK3CA* fragments obtained from MDA‐MB‐231 cells that contain the wild‐type fragments (E545) were used instead of healthy individuals, because the corresponding target region could not be amplified by PCR from isolated ctDNAs obtained from healthy individuals, possibly due to very low release of ctDNA into the blood or effective fragmentation of the target region in healthy individuals. Overall, these results indicate that our PER‐based detection approach can specifically detect the single mutations in target RNA strands on lateral flow strips. Our results open new possibilities for clinical applications. For instance, this test could be used to closely monitor actionable mutations in ctDNA over time, both during and after therapy. It can be conveniently implemented in routine molecular diagnostic laboratories outside specialized centers, serving as an alternative to DNA sequencing that is substantially faster and available at a fraction of the cost.

## 3 Conclusion

In conclusion, we developed a rapid, sensitive, and highly specific PER‐based signal amplification strategy for the detection of cancer‐specific mutations using LFAs. Our system enables precise detection of both synthetic ODNs and endogenous nucleic acids containing cancer‐relevant mutations, extracted from human cells and plasma of breast cancer patients. The detection relies on a one‐step, self‐assembly reaction completed in just 10 min, and the entire workflow—including preamplification, RNA synthesis, and cleavage steps—can be completed within 5–6 h. By employing PER‐based linear signal amplification, we achieved a 16‐fold increase in sensitivity compared to conventional single‐dye labeling, reaching a detection limit as low as 16 pM. Notably, our approach enables the detection of nucleic acids in various biological fluids including serum and saliva. Using a dual‐target LFA format, we successfully identified mutant strands even in the presence of a 10‐fold excess of wild‐type sequences. Importantly, our platform demonstrated precise discrimination of single‐nucleotide mutations commonly found in breast cancer, underscoring its potential for rapid, POC mutation screening. Furthermore, by preamplifying low abundant ctDNAs from breast cancer patient plasma, we detected clinically relevant single‐nucleotide mutations in the *PIK3CA* gene. This highlights the potential of our system as a rapid screening alternative to conventional sequencing techniques. The programmability of our DNA‐based method also facilitates straightforward probe‐design and scalability, providing the foundation for future multiplexed detection of multiple mutations within a single assay. Altogether, these results show the potential of our biosensor to be used in laboratories for research purposes and in the clinics for rapid screening and monitoring of cancer‐related mutations, companion diagnostics for personalized cancer therapies, and disease monitoring. In a broader perspective, our approach can be adapted to detect mutations associated with other genetic disorders and to expand the capabilities of conventional LFAs for nucleic acid diagnostics.

## 4 Experimental Section

### 4.1 Materials

All unmodified and biotin‐modified DNA ODNs were ordered from LubioScience‐Switzerland IDT (Zurich, Switzerland). FITC and digoxigenin‐modified ODNs (HPLC purified) were purchased from Biomers GmbH (Ulm, Germany). The detailed sequences of the ODNs are given in Table S1. Lateral flow assay stripes (HybriDetect 2T) were obtained from Milenia Biotech GmbH (Giesen, Germany). Taq DNA polymerase (cat: 10342053), SYBR safe DNA gel stain (10×, cat: S33102), GeneRuler DNA ladder mix (cat: SM0331), and all cell culture reagents were ordered from Thermo Fisher Scientific (Basel, Switzerland). Bst DNA polymerase (M0275L, 8000  U/mL), MgSO_4_ (100 mM), ThermoPol reaction buffer, deoxynucleotide (dNTP) solution mix (N0447S), RNase H (M0297S), HiScribe T7 High Yield RNA Synthesis Kit (E2040S) and Monarch RNA Cleanup Kit (T2050L, 500 μg) were purchased from New England Biolabs (NEB) (Ipswich, MA, USA). NucleoSpin Gel and PCR Clean‐up kit (740609.50), cfDNA isolation kit (cfDNA XS, 740900.50) and RNA isolation kit (NucleoSpin RNA plus) were purchased from Macherey‐Nagel.

### 4.2 Preparation of PER Concatamers

PER concatemers were prepared in 100 μL of reaction mix, including 1× ThermoPol reaction buffer (diluted from 10× stock) with final concentrations of 10 mM MgSO4, 400  U/mL of Bst LF polymerase, 600 μM each of dATP, dCTP, and dTTP, 100 nM of Clean G hairpin and 0.15 μM of hairpin for target ODN. After addition of water to 99 μL, and the reaction mixture was incubated for 15 min at 37°C, followed by the addition of 1 μL of 100 μM primer (Xp) to obtain 1 μM final concentration, and the reaction was incubated for another 3 h at 37°C. The reaction was terminated by heating to 80°C for 20 min to deactivate the polymerase.

### 4.3 Isolation of RNAs from Human Cells and cfDNAs from Human Plasma

Human cancer cell lines (MCF‐7, MDA‐MB‐231) were cultured at 37°C, 5% CO_2_, and 95% humidity in Dulbecco's modified Eagle's medium (DMEM) supplemented with Glutamax, 10% fetal bovine serum, and 1% penicillin and streptomycin. mRNA isolation from these cell lines was performed using the NucleoSpin RNA plus RNA isolation kit (10^6^ cells). Extraction was done as described in the kit protocol. To isolate circulating DNAs from human blood samples, 240 μL of plasma was used for each patient sample (12 samples from breast cancer patients). Previously collected plasma samples were used in this study [34]. Ethical approval was obtained from the Cantonal Ethic Commission for Human Research on Humans of the Canton Ticino and extended to Vaud–Fribourg–Neuchatel (Switzerland) (ce 2967 BASEC PB 2017‐00207).

### 4.4 CDNA Synthesis, PCR, IVT and RNase H Cleavage

Isolated RNA samples were reversely transcribed into cDNAs using the High‐Capacity cDNA Reverse Transcription kit. First, 10 μL of 2× RT master mix was prepared according to the kit protocol and mixed with 10 μL (1 μg) of RNA isolate from cells for each sample. Samples were then incubated in a thermal cycler (Biometra TAdvanced, Analytik Jena) at 37°C for 2 h followed by 85°C for 5 min to inactivate the enzymes, and immediately cooled down to 4°C. For the amplification of target gene regions from cDNAs and plasma isolated cfDNAs, 2 μL of cDNA template or cfDNA was mixed with 2 μL of 10× Taq polymerase reaction buffer, 0.8 μL of MgCl_2_ (2 mM), 0.4 μL of dNTP mix (200 μM), 0.2 μL of forward primer with T7 RNA polymerase binding site (20 μM), 0.4 μL of reverse primer (10 μM), 0.2 μL of Taq DNA polymerase, and 14 μL of RNase‐free water for PCR reaction. Samples were incubated in the thermal cycler with a temperature profile of 95°C for 3 min, followed by 35 or 40 amplification cycles (95°C for 20 s, 57°C for 40 s, and 72°C for 40 s), and a final extension at 72°C for 10 min. The primer sequence is provided in Table S1.

To transcribe target genes (*PIK3CA* and *P53*) from amplified PCR products, IVT was applied by mixing 2 μL of PCR product with 2 μL of 10x reaction buffer, 8 μL of NTP mix (ATP, GTP, CTP, UTP, 10 mM each), 1 μL of DTT (5 mM), and 2 μL of T7 RNA polymerase mix and completed up to 20 μL with RNase free water as described in the protocol of HiScribe T7 High Yield RNA Synthesis Kit. The solution was incubated in the thermal cycler at 37°C for 2 h. After incubation, synthesized RNA was purified using Monarch RNA Cleanup Kit.

Finally, RNAse H enzyme was used for the fragmentation of transcribed RNAs. For this, guide ODNs (20 nt single‐stranded DNA) were first designed to bind upstream and downstream on the specific regions in the target RNA sequence. Then, transcribed RNA sample (10 μg) was mixed with 4 μL of guide ODNs (1 μM each), completed up to 35 μL with RNase free water, and heated it to 70°C for 5 min for DNA:RNA hybridization. Then, 4 μL of 10× RNAse H reaction buffer and 1 μL of RNase H was added, mixed and heated for 2 h at 37°C to allow the enzyme to cut RNA in the DNA:RNA hybrid for the release of the target RNA for detection. Finally, RNase H was inactivated by incubation at 65°C for 10 min.

### 4.5 Detection of Target DNAs, RNA Fragments and Mutations by Lateral Flow Assay

The LFAs were performed using HybriDetect 2T test strips. All strips contained a nitrocellulose membrane coated with streptavidin and polyclonal anti‐digoxigenin antibody (goat) test lines and polyclonal anti‐goat antibody (rabbit) control line. For running the LFAs for the detection of target ODNs (ODNs), the detection mixture was first prepared by mixing 10 μL of biotinylated ODN (100 nM), 10 μL of PER concatemer (100 nM), 1 μL of FITC‐labeled ODN (1 μM), target ODN with desired concentration and completed up to 100 μL with 1× PBS. For the detection of mutations (as in Figures 3 and 4), 10 μL of digoxigenin‐labeled ODN (100 nM) was also added to the detection mixture in addition to the biotinylated ODN. The mixture was briefly vortexed and directly applied on the lateral flow strips. For that, 20 μL of detection mixture was further mixed with 80 μL of running buffer in a well of 96‐well plate and flow strips were dipped into the solution through the SAP. 10 min after incubation, the strips were imaged using iPhone 15 camera. The band intensities were further analyzed by ImageJ using the Plot Lanes tool (Analyze/Gels/Plot Lanes). After defining the first lane and selecting the bands with the rectangular selection tool, each subsequent lane is marked in the same way to generate a profile that visually represents the intensity and size of each band. The area of all the peaks above the baseline were measured using wand tool and depicted as intensity (in Figure 2, 3, and 4). The collected intensity versus concentration data in Figure 2 was observed to follow symmetrical and sigmoidal trends, which could be fitted with a nonlinear 4‐parameter logistic function,

(1) $$Y=a+\frac{(d-a)X^{b}}{X^{b}+c^{b}}$$

where *a* is the lower asymptote (bottom), *d* is the upper asymptote (top), *c* is the inflection point of the curve and *b* is the Hillslope. The non‐linear curve fitting was performed with GraphPad Prism version 10.5.0.

Human serum (from male AB clotted whole blood, Sigma–Aldrich, H6914) and human saliva (collected from healthy volunteers) were used instead of 1× PBS in the detection mixture in the experiment shown in the Figure 2. For the detection of RNAse H cleaved target RNA fragments carrying mutations using lateral flow strips (as in Figure 4), all or half of the 40 μL of the reaction mix (see the previous section for the preparation) was directly added into the detection mix and then mixed with running buffer which resulted in 1–2 μg final RNA concentration.

Statistical Analysis: All data presented in this study (Figures 2A,B, 3B and 4D) were expressed as the mean  ±  SD of *n* = 3 independent experiments, as stated also in the corresponding Figure captions.

## Supporting Information

Additional supporting information can be found online in the Supporting Information section. **Supporting Fig. S1:** Agarose gel analysis of PER concatamers. The PER solution was incubated for 3 h at 37°C. After heat inactivation of polymerase for 20 min, samples were mixed with 6x loading dye and run in 1.5% agarose containing 1x SYBR safe at 80 V for 40 min. 20 μL of unpurified solution out of 100 μL PER assembly was loaded into the gel in the presence or absence of imager strand (IS‐647). 100 pmol primer and 100 pmol hairpin were loaded as controls. **Supporting Fig. S2:** Detection of P53 fragments using gold nanoparticles of various sizes and branched signal amplification. A) Detection of P53 fragments (WT: R280, M: K280) using 5 nm and 10 nm AuNPs. In order to selective capture of these target ODNs, we used biotinylated ODN for the R280 fragment and digoxigenin‐labeled ODN for the K280 fragment. We combined these probes (100 nM) with PER‐generated concatemers (100 nM), FITC‐labeled imager strands (500 nM) and the target fragments (left: WT or mutant P53, 10 nM, right: 1 nM to 1 pM) and completed up to 90 μL with 1xPBS. Then, the solution was mixed with 10 μL of 5 nm or 10 nm anti‐FITC‐AuNP (0.15 mg/mL at 3 OD, Cytodiagnostics, CYDI‐AC‐5‐20 or CYDI‐AC‐10‐20). Following a brief incubation, 20 μL of the solution was combined with 80 μL of running buffer in a well of a 96‐well plate, into which custom flow strips (Attogene) were subsequently inserted. B) Concentration‐dependent detection of WT *P53* fragments on lateral flow strips (Milenia Biotech) using linear and branched PER signal amplification. **Supporting Fig. S3:** PCR amplification of target gene regions (*P53*, *PIK3CA*, *ESR1*) from ctDNAs extracted from breast cancer patient plasma. 10 μL of amplicon was mixed with 6x loading dye and run in 1.5% agarose containing 1x SYBR safe at 70 V for 45 min. The lengths of the amplified regions (including T7 polymerase binding region) are: A) P53: 331 bp, B) PIK3CA: 291 bp, C) ESR1: 277 bp. **Supporting Fig. S4:** The alignment results of target gene regions (*P53*, *PIK3CA*, *ESR1*) amplified from ctDNAs extracted from breast cancer patient plasma. FASTA sequences obtained via Sanger sequencing were aligned using ApE software and screened mutations were highlighted in dashed rectangles. (*P53*: R273, R280; *PIK3CA*: E542, E545; *ESR1*: Y537, D538) The nucleotide signal intensities of the screened PIK3CA E545 mutation, obtained by Sanger sequencing of the amplicons, demonstrated a heterozygous E545A (A/C) genotype. **Supporting Table S1:** List of oligonucleotide sequences. **Supporting Table S2:** The sequences of amplified regions from cell extracted RNAs.

## Conflicts of Interest

S. K. and C. R. declare the following competing financial interest: A patent application (PCT/EP EP24202960.1; Methods of Detection of Target RNAs in a sample and uses thereof) has been submitted. C. R. is co‐founder and owns shares of Xemperia.

## Supporting information

Supplementary Material

## Data Availability

The data that support the findings of this study are available within this article and its Supporting Information and from the corresponding author(s) upon request.

## References

[1] N. Sinha and R. Nussinov , “Point Mutations and Sequence Variability in Proteins: Redistributions of Preexisting Populations,” Proceedings of the National Academy of Sciences of the United States of America 98, no. 6 (2001): 3139–3144, 10.1073/pnas.051399098. 11248045 PMC30620

[2] P. J. Stephens , P. S. Tarpey , H. Davies , et al., “The Landscape of Cancer Genes and Mutational Processes in Breast Cancer,” Nature 486, no. 7403 (2012): 400–404, 10.1038/nature11017. 22722201 PMC3428862

[3] J. Lilyquist , K. J. Ruddy , C. M. Vachon , and F. J. Couch , “Common Genetic Variation and Breast Cancer Risk‐Past, Present, and Future,” Cancer Epidemiology, Biomarkers & Prevention 27, no. 4 (2018): 380–394, 10.1158/1055-9965.EPI-17-1144. PMC5884707 29382703

[4] M. L. Tornesello , “TP53 Mutations in Cancer: Molecular Features and Therapeutic Opportunities (Review),” International Journal of Molecular Medicine 55, no. 1 (2025): 7, 10.3892/ijmm.2024.5448. 39450536 PMC11554381

[5] M. Davies , B. Hennessy , and G. B. Mills , “Point Mutations of Protein Kinases and Individualised Cancer Therapy. *Expert Opin Pharmacother* ,” Expert Opinion on Pharmacotherapy 7, no. 16 (2006): 2243–2261, 10.1517/14656566.7.16.2243 17059381

[6] H. Schwarzenbach , D. S. Hoon , and K. Pantel , “Cell‐Free Nucleic Acids as Biomarkers in Cancer Patients,” Nature Reviews Cancer 11, no. 6 (2011): 426–437, 10.1038/nrc3066. 21562580

[7] D. S. Guttery , K. Page , A. Hills , et al., “Noninvasive Detection of Activating Estrogen Receptor 1 (ESR1) Mutations in Estrogen Receptor‐Positive Metastatic Breast Cancer,” Clinical Chemistry 61, no. 7 (2015): 974–982, 10.1373/clinchem.2015.238717. 25979954

[8] A. Tadimety , Y. Zhang , K. M. Kready , et al., “Design of Peptide Nucleic Acid Probes on Plasmonic Gold Nanorods for Detection of Circulating Tumor DNA Point Mutations,” Biosensors & Bioelectronics 130 (2019): 236–244, 10.1016/j.bios.2019.01.045. 30769288

[9] S. Hassan , A. Shehzad , S. A. Khan , et al., “Diagnostic and Therapeutic Potential of Circulating‐Free DNA and Cell‐Free RNA in Cancer Management. *Biomedicines* ,” Biomedicines 10, no. 8 (2022): 2047, 10.3390/biomedicines10082047. 36009594 PMC9405989

[10] M. Elazezy and S. A. Joosse , “Techniques of Using Circulating Tumor DNA as a Liquid Biopsy Component in Cancer Management,” Computational and Structural Biotechnology Journal 16 (2018): 370–378, 10.1016/j.csbj.2018.10.002. 30364656 PMC6197739

[11] S. Shin , S. Han , J. Kim , Y. Shin , J.‐J. Song , and S. Hohng , “Fast, Sensitive, and Specific Multiplexed Single‐Molecule Detection of Circulating Tumor DNA,” Biosensors and Bioelectronics 242 (2023): 115694, 10.1016/j.bios.2023.115694. 37797531

[12] P. Song , L. R. Wu , Y. H. Yan , et al., “Limitations and Opportunities of Technologies for the Analysis of Cell‐Free DNA in Cancer Diagnostics,” Nature Biomedical Engineering 6, no. 3 (2022): 232–245, 10.1038/s41551-021-00837-3. PMC9336539 35102279

[13] L. Fancello , S. Gandini , P. G. Pelicci , and L. Mazzarella , “Tumor Mutational Burden Quantification from Targeted Gene Panels: Major Advancements and Challenges,” Journal for Immunotherapy of Cancer 7, no. 1 (2019): 183, 10.1186/s40425-019-0647-4. 31307554 PMC6631597

[14] B. Leatham , K. McNall , H. K. K. Subramanian , et al., “A Rapid, Multiplex Digital PCR Assay to Detect Gene Variants and Fusions in Non‐Small Cell Lung Cancer,” Molecular Oncology 17, no. 11 (2023): 2221–2234, 10.1002/1878-0261.13523. 37714814 PMC10620117

[15] S. Kakkar , P. Gupta , S. P. Singh Yadav , et al., “Lateral Flow Assays: Progress and Evolution of Recent Trends in Point‐of‐Care Applications,” Materials Today Bio 28 (2024): 101188, 10.1016/j.mtbio.2024.101188. PMC11364909 39221210

[16] A. Sena‐Torralba , R. Alvarez‐Diduk , C. Parolo , A. Piper , and A. Merkoci , “Toward Next Generation Lateral Flow Assays: Integration of Nanomaterials,” Chemical Reviews 122, no. 18 (2022): 14881–14910, 10.1021/acs.chemrev.1c01012. 36067039 PMC9523712

[17] D. Quesada‐Gonzalez and A. Merkoci , “Nanoparticle‐Based Lateral Flow Biosensors,” Biosensors & Bioelectronics 73 (2015): 47–63, 10.1016/j.bios.2015.05.050. 26043315

[18] C. Parolo and A. Merkoci , “Paper‐Based Nanobiosensors for Diagnostics,” Chemical Society Reviews 42, no. 2 (2013): 450–457, 10.1039/c2cs35255a. 23032871

[19] Y. Liu , L. Zhan , Z. Qin , J. Sackrison , and J. C. Bischof , “Ultrasensitive and Highly Specific Lateral Flow Assays for Point‐of‐Care Diagnosis,” ACS Nano 15, no. 3 (2021): 3593–3611, 10.1021/acsnano.0c10035. 33607867

[20] K. Sun , X. Wang , Y. Qu , H. Wang , and J. Cheng , “Amplification‐Free Nucleic Acid Testing with a Fluorescence One‐Step‐Branched DNA‐Based Lateral Flow Assay (FOB‐LFA),” Analytical Chemistry 95, no. 36 (2023): 13605–13613, 10.1021/acs.analchem.3c02299. 37594225

[21] S. J. Schulte , J. Huang , and N. A. Pierce , “Hybridization Chain Reaction Lateral Flow Assays for Amplified Instrument‐Free At‐Home SARS‐CoV‐2 Testing,” ACS Infectious Diseases 9, no. 3 (2023): 450–458, 10.1021/acsinfecdis.2c00472. 36735927 PMC9924079

[22] H. Ijas , J. Trommler , L. Nguyen , et al., “DNA Origami Signal Amplification in Lateral Flow Immunoassays,” Nature Communications 16, no. 1 (2025): 3216, 10.1038/s41467-025-57385-6. PMC11971289 40185718

[23] S. Umrao , M. Zheng , X. Jin , S. Yao , and X. Wang , “Net‐Shaped DNA Nanostructure‐Based Lateral Flow Assays for Rapid and Sensitive SARS‐CoV‐2 Detection,” Analytical Chemistry 96, no. 8 (2024): 3291–3299, 10.1021/acs.analchem.3c03698. 38306661 PMC10922791

[24] H. N. Lee , J. Lee , Y. K. Kang , J. H. Lee , S. Yang , and H. J. Chung , “A Lateral Flow Assay for Nucleic Acid Detection Based on Rolling Circle Amplification Using Capture Ligand‐Modified Oligonucleotides,” BioChip Journal 16, no. 4 (2022): 441–450, 10.1007/s13206-022-00080-1. 36091642 PMC9446602

[25] M. Jauset‐Rubio , M. Svobodova , T. Mairal , et al., “Ultrasensitive, Rapid and Inexpensive Detection of DNA Using Paper Based Lateral Flow Assay,” Scientific Reports 6 (2016): 37732, 10.1038/srep37732. 27886248 PMC5123575

[26] S. Agarwal , M. Hamidizadeh , and F. F. Bier , “Detection of Reverse Transcriptase LAMP‐Amplified Nucleic Acid from Oropharyngeal Viral Swab Samples Using Biotinylated DNA Probes through a Lateral Flow Assay,” Biosensors 13, no. 11 (2023): 988, 10.3390/bios13110988. 37998163 PMC10669123

[27] M. Huang , Y. Xiang , Y. Chen , et al., “Bottom‐Up Signal Boosting with Fractal Nanostructuring and Primer Exchange Reaction for Ultrasensitive Detection of Cancerous Exosomes,” ACS Sensors 8, no. 3 (2023): 1308–1317, 10.1021/acssensors.2c02819. 36855267

[28] J. Y. Kishi , S. W. Lapan , B. J. Beliveau , et al., “SABER Amplifies FISH: Enhanced Multiplexed Imaging of RNA and DNA in Cells and Tissues,” Nature Methods 16, no. 6 (2019): 533–544, 10.1038/s41592-019-0404-0. 31110282 PMC6544483

[29] S. K. Saka , Y. Wang , J. Y. Kishi , et al., “Immuno‐SABER Enables Highly Multiplexed and Amplified Protein Imaging in Tissues,” Nature Biotechnology 37, no. 9 (2019): 1080–1090, 10.1038/s41587-019-0207-y. PMC6728175 31427819

[30] S. Kocabey , S. Cattin , I. Gray , and C. Ruegg , “Ultrasensitive Detection of Cancer‐Associated Nucleic Acids and Mutations by Primer Exchange Reaction‐Based Signal Amplification and Flow Cytometry,” Biosensors & Bioelectronics 267 (2025): 116839, 10.1016/j.bios.2024.116839. 39369516

[31] T. Shimoi , A. Hamada , M. Yamagishi , et al., “PIK3CA Mutation Profiling in Patients with Breast Cancer, Using a Highly Sensitive Detection System,” Cancer Science 109, no. 8 (2018): 2558–2566, 10.1111/cas.13696. 29906308 PMC6113507

[32] A. Gomes , F. Trovão , B. Andrade Pinheiro , et al., “The Crystal Structure of the R280K Mutant of Human p53 Explains the Loss of DNA Binding,” International Journal of Molecular Sciences 19, no. 4 (2018): 1184, 10.3390/ijms19041184. 29652801 PMC5979565

[33] F. Bošković , J. Zhu , R. Tivony , et al., “Simultaneous Identification of Viruses and Viral Variants with Programmable DNA Nanobait,” Nature Nanotechnology 18, no. 3 (2023): 290–298, 10.1038/s41565-022-01287-x. PMC10020084 36646828

[34] S. Cattin , B. Fellay , A. Calderoni , et al., “Circulating Immune Cell Populations Related to Primary Breast Cancer, Surgical Removal, and Radiotherapy Revealed by Flow Cytometry Analysis,” Breast Cancer Research 23, no. 1 (2021): 64, 10.1186/s13058-021-01441-8. 34090509 PMC8180078

