# Supplementary material for: Detection of Cancer‐Associated Mutations Using Primer Exchange Reaction‐Based Signal Amplification and Lateral Flow Assays
Source: Small Sci. 2026 Feb 6;6(2):e202500520. doi: 10.1002/smsc.202500520 (PMC12884750; doi:10.1002/smsc.202500520)
Supplement: Supplementary file 1 — Supplementary Material [file SMSC-6-e202500520-s001.pdf]

Supporting Information

Detection of Cancer-Associated Mutations using Primer Exchange Reaction-Based Signal Amplification and Lateral Flow Assays

*Samet Kocabey * [a,b] and Curzio Rüegg * [b,c]*

a Laboratory of Experimental and Translational Oncology, Department of Oncology, Microbiology and Immunology, Faculty of Science and Medicine, University of Fribourg, Chemin du Musée 18, PER17, 1700 Fribourg, Switzerland

b NCCR Bio-inspired Materials, University of Fribourg, 1700 Fribourg, Switzerland

c Xemperia SA, Marly Innovation Centre, Route de l`Ancienne Papeterie, 1723, Marly, Switzerland

* Corresponding authors

E-mail: samet.kocabey@unifr.ch, curzio.ruegg@unifr.ch

Table of Contents

1. Supporting Tables 1

Table S1. List of oligonucleotide sequences........ 1

Table S2. The sequences of amplified regions from cell extracted RNAs 2

2. Supporting Figures........ 3

Figure S1. Agarose gel analysis of PER concatamers........ 3

Figure S2. Detection of P53 fragments using gold nanoparticles of various sizes and branched signal amplification. 4

Figure S3. PCR amplification of target gene regions (*P53*, *PIK3CA*, *ESR1*) from ctDNAs extracted from breast cancer patient plasma. 5

Figure S4. The alignment results of target gene regions (*P53*, *PIK3CA*, *ESR1*) amplified from ctDNAs extracted from breast cancer patient plasma. 6

1. Supporting Tables

Table S1. List of oligonucleotide sequences

Probe name	Sequences (5`-3`)	Modification
**Targets and Probe oligonucleotides**		
P53-R280	TGTCCTGGGAGAGACCGG	
P53-K280	TGTCCTGGGAAAGACCGG	
R280-Biotin	TTTTTTCCGGTCTCT	5` Biotin
K280-Digo	TTTTTTCCGGTCTTT	5` Digoxigenin
PIK3CA-E545	TCTCTCTGAAATCACTGAGCA	
PIK3CA-K545	TCTCTCTGAAATCACTAAGCA	
PIK3CA-A545	TCTCTCTGAAATCACTGCGCA	
E545-Biotin	TTTTTGCTCAGTGAT	5` Biotin
K545-Digo	TTTTTGCTTAGTGAT	5` Digoxigenin
A545-Digo	TTTTTGCGCAGTGAT	5` Digoxigenin
**Oligonucleotides used for Primer Exchange Reaction**		
P53-Linear-Primer	CCCAGGACATTCCAATAATA	
PIK3CA-Linear-Primer	TTCAGAGAGATTCCAATAATA	
Branching Primer	GATTTTAGATGATTTTAGAT GATTTTAGATTTCCAATAATA	
Hairpin-25 (for Linear amplification)	ACCAATAATAGGGCCTTTTGGCCC TATTATTGGTTATTATTGGT	3` Inverted dT
Hairpin-29 (for Branch amplification)	ATCTAAAATCGGGCCTTTTGGCCC GATTTTAGATGATTTTAGAT	3` Inverted dT
Clean G Hairpin	CCCCGAAAGTGGCCTCGGGCCTTTTGGCCC GAGGCCACTTTCG	
Imager-FITC	TTTATTATTGGTTATTATTGGT	5` FITC
**Primers used for PCR and in vitro transcription**		
T7-PIK3CA-G (Forward)	TAATACGACTCACTATAGGG TCATCTGTGAATCCAGAGGGGA	
PIK3CA-G (Reverse)	ATGCTGAGATCAGCCAAATTCAGT	
T7-P53-G (Forward)	TAATACGACTCACTATAGGG AAATGGGACAGGTAGGACCTGA	
P53-G (Reverse)	TGGTGTTGTTGGGCAGTGC	
T7-ESR1-G (Forward)	TAATACGACTCACTATAGGG CCTTCCCCTTCTAGGGATTTCAGC	
ESR1-G (Reverse)	GCGATGAAGTAGAGCCCGCA	
**Guide oligonucleotides for RNAse H cleavage**		
PIK3CA-guide1	GGATCTCGTGTAGAAATTGC	
PIK3CA-guide2	TCCATAGAAAATCTTTCTCC	
P53-guide1	GGCACAAACACGCACCTCAA	
P53-guide2	AGATTCTCTTCCTCTGTGCG	

The nomenclature in Figure 1 and corresponding sequences in Table S1:

x: the first part of any primer for linear PER that is complementary to the target DNA or RNA sequence (e.g. P53-Linear-Primer: CCCAGGACA)

p: extended region by PER & the repetitive segments in the linear PER concatemer (CCAATAATA)

p*: complementary region for p that is found in the Hairpin-25 and Imager-FITC (TATTATTGG)

Table S2. The sequences of amplified regions from cell extracted RNAs

PIK3CA-MCF7	ACTATTCCCACGCAGGACTGAGTAACAGACTAGCTAGAGACAATGAATTAAGGGAAAATGAC<br>AAAGAACAGCTCAAAGCAATTTCTACACGAGATCCTCTCTCTGAAATCACTRAGCAGGAGAA<br>AGATTTTCTATGGAGTCACAGACACTATTGTGTAACTATCCCCGAAATTCTACCCAAATTGCTT<br>CTGTCTGTTAAATGGAATTCTAGAGATGAAGTAGCCCAGATGTATTGCTTGGTAAAAGATTGS<br>CCCTCCAATCAAATS
PIK3CA-MDA231	TWRMTATTCCCACGCAGGACTGAGTAACAGACTAGCTAGAGACAATGAATTAAGGGAAAAT<br>GACAAAGAACAGCTCAAAGCAATTTCTACACGAGATCCTCTCTCTGAAATCACTGAGCAGGA<br>GAAAGATTTTCTATGGAGTCACAGACACTATTGTGTAACTATCCCCGAAATTCTACCCAAATT<br>GCTTCTGTCTGTTAAATGGAATTCTAGAGATGAAGTAGCCCAGATGTATTGCTTGGTAAAAG<br>ATTGC
P53-MCF7	KGGCTCTGACTGTACCACCATCCACTACAACTACATGTGTAACAGTTCCTGCATGGGCGGCAT<br>GAACCGGAGGCCCATCCTCACCATCATCACACTGGAAGACTCCAGTGGTAATCTACTGGGAC<br>GGAACAGCTTTGAGGTGCGTGTTTGTGCCTGTCCTGGGAGAGACCGGCGCACAGAGGAAGA<br>GAATCTCCGCAAGAAAGGGGAGCCTCACCACGAGCTGCCCCCAGGGAGCACTAAGCGAGCA<br>CTGCCCAACAACACCAAAGC
P53-MDA231	GCTCTGACTGTACCACCATCCACTACAACTACATGTGTAACAGTTCCTGCATGGGCGGCATGA<br>ACCGGAGGCCCATCCTCACCATCATCACACTGGAAGACTCCAGTGGTAATCTACTGGGACGG<br>AACAGCTTTGAGGTGCGTGTTTGTGCCTGTCCTGGGAAAGACCGGCGCACAGAGGAAGAGA<br>ATCTCCGCAAGAAAGGGGAGCCTCACCACGAGCTGCCCCCAGGGAGCACTAAGCGAGCACT<br>GCCCAACAACACCAATTKTTC

Binding sites for guide oligonucleotides   Mutation codon

2. Supporting Figures

Figure S1. Agarose gel analysis of PER concatamers

The PER solution was incubated for 3 h at 37 °C. After heat inactivation of polymerase for 20 min, samples were mixed with 6x loading dye and run in 1.5 % agarose containing 1x SYBR safe at 80 V for 40 min. 20 μL of unpurified solution out of 100 μL PER assembly was loaded into the gel in the presence or absence of imager strand (IS-647). 100 pmol primer and 100 pmol hairpin were loaded as controls.

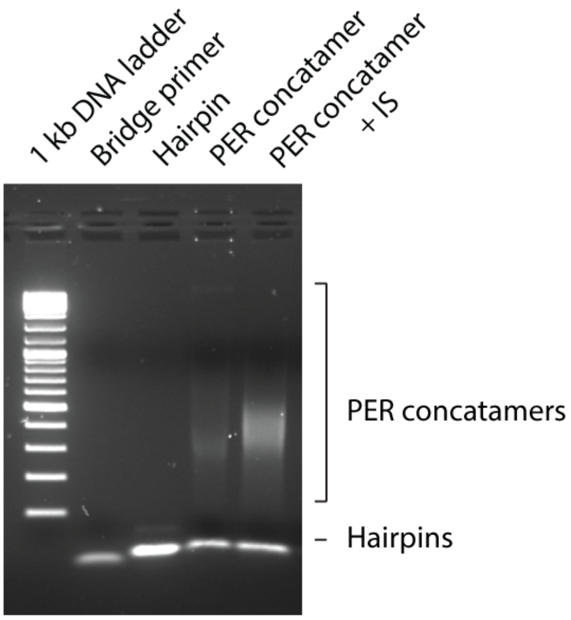

Figure S2. Detection of P53 fragments using gold nanoparticles of various sizes and branched signal amplification.

A) Detection of P53 fragments (WT: R280, M: K280) using 5 nm and 10 nm AuNPs. In order to selective capture of these target ODNs, we used biotinylated ODN for the R280 fragment and digoxigenin-labeled ODN for the K280 fragment. We combined these probes (100 nM) with PER-generated concatemers (100 nM), FITC-labeled imager strands (500 nM) and the target fragments (left: WT or mutant P53, 10 nM, right: 1 nM to 1 pM) and completed up to 90 μL with 1xPBS. Then, the solution was mixed with 10 μL of 5 nm or 10 nm anti-FITC-AuNP (0.15 mg/mL at 3 OD, Cytodiagnostics, CYDI-AC-5-20 or CYDI-AC-10-20). Following a brief incubation, 20 μL of the solution was combined with 80 μL of running buffer in a well of a 96-well plate, into which custom flow strips (Attogene) were subsequently inserted.

B) Concentration-dependent detection of WT *P53* fragments on lateral flow strips (Milenia Biotech) using linear and branched PER signal amplification.

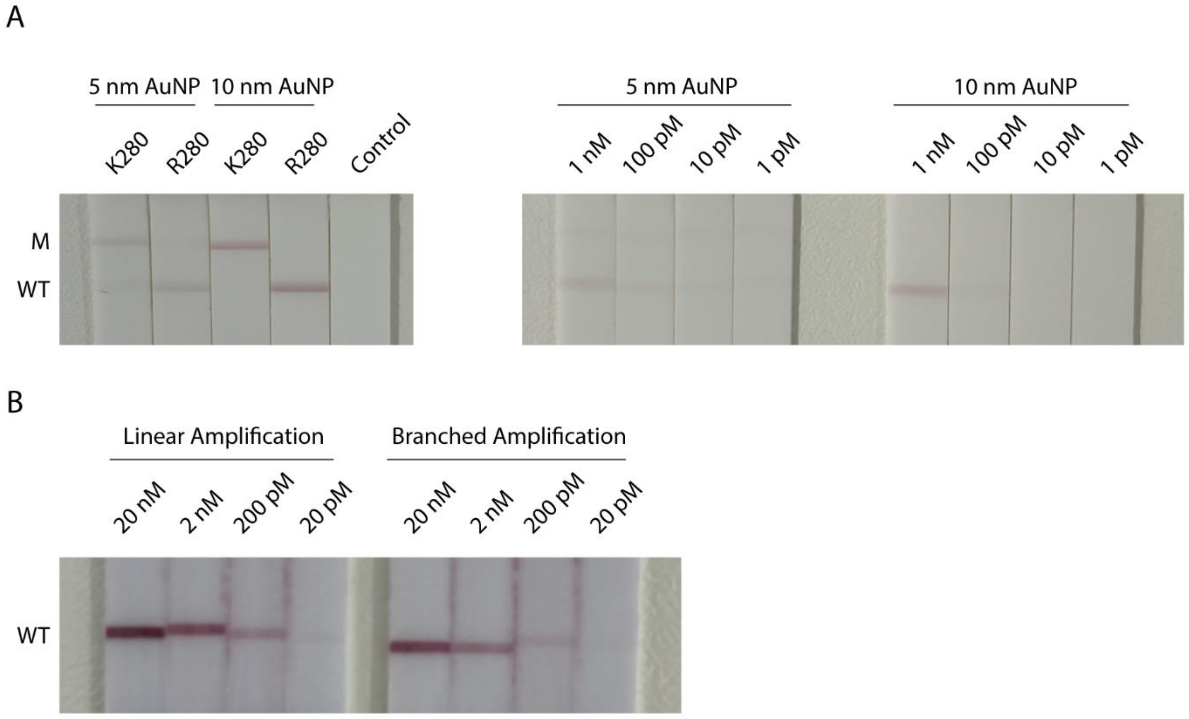

Figure S3. PCR amplification of target gene regions (*P53*, *PIK3CA*, *ESR1*) from ctDNAs extracted from breast cancer patient plasma.

10 μL of amplicon was mixed with 6x loading dye and run in 1.5 % agarose containing 1x SYBR safe at 70 V for 45 min. The length of amplified regions (including T7 polymerase binding region are): A) P53: 331 bp, B) PIK3CA: 291 bp, C) ESR1: 277 bp

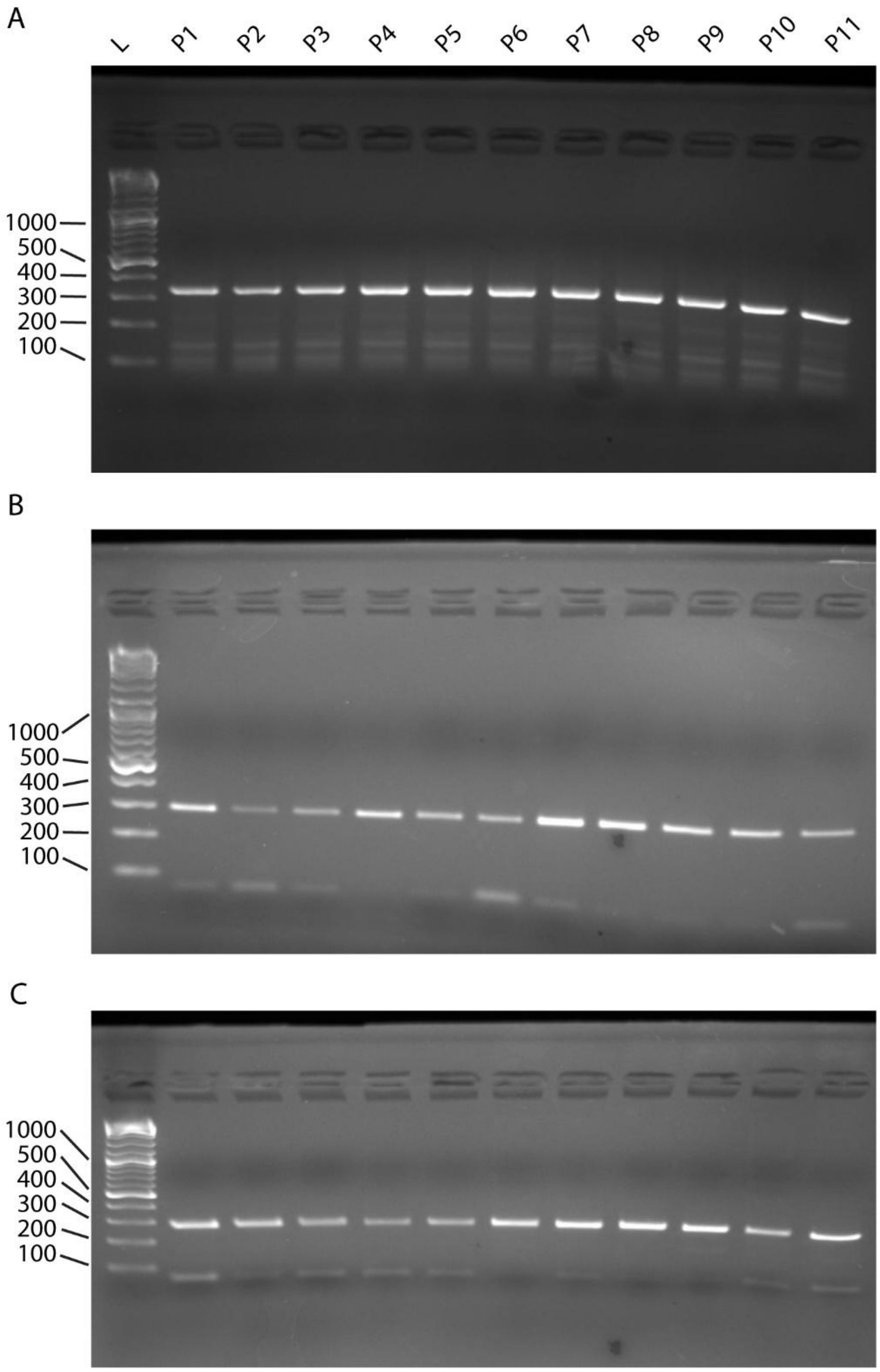

Figure S4. The alignment results of target gene regions (*P53*, *PIK3CA*, *ESR1*) amplified from ctDNAs extracted from breast cancer patient plasma.

FASTA sequences obtained via Sanger sequencing were aligned using ApE software and screened mutations were highlighted in dashed rectangles. (*P53*: R273, R280; *PIK3CA*: E542, E545; *ESR1*: Y537, D538) The nucleotide signal intensities of the screened PIK3CA E545 mutation, obtained by Sanger sequencing of the amplicons, demonstrated a heterozygous E545A (A/C) genotype.

```
Wed May 28, 2025 17:08 CEST
BC-P53-012.fasta from 1 to 251
Alignment to
BC-P53-001.fasta--  Matches:247; Mismatches:4; Gaps:0
BC-P53-002.fasta--  Matches:246; Mismatches:3; Gaps:0
BC-P53-003.fasta--  Matches:248; Mismatches:3; Gaps:0
BC-P53-004.fasta--  Matches:250; Mismatches:0; Gaps:0
BC-P53-005.fasta--  Matches:250; Mismatches:1; Gaps:0
BC-P53-006.fasta--  Matches:251; Mismatches:0; Gaps:0
BC-P53-007.fasta--  Matches:251; Mismatches:0; Gaps:0
BC-P53-008.fasta--  Matches:250; Mismatches:1; Gaps:0
BC-P53-009.fasta--  Matches:251; Mismatches:0; Gaps:0
BC-P53-010.fasta--  Matches:251; Mismatches:0; Gaps:0
BC-P53-011.fasta--  Matches:251; Mismatches:0; Gaps:0

                                                                   R273                    R280
             *         *         *         *         *         *         *         *         *         *
  1>~~~~~~~~~~~~~~~~~~~~~~~~~~~~~~~TARTGGTAATCTACTGGGACGGAACAGCTTTGAGGTGCGTGTTTGTGCCTGTCCTGGGAGAGACCGGCG>69
  1>~~~~~~~~~~~~~~~~~~~~~~~~~~~~~~~TARKGGTAATCTACTGGGACGGAACAGCTTTGAGGTGCGTGTTTGTGCCTGYCCWGGGARAGACCGGCG>69
  1>~~~~~~~~~~~~~~~~~~~~~~~~~~~~~~~~~RKGGTAATCTACTGGGACGGAMCAGCTTTGAGGTGCGTGTTTGTGCCTGYCCTGGGAGAGACCGGCG>67
  1>G------------------------------TARKGGTAATCTACTGGGACGGAACAGCTTTGAGGTGCGTGTTTGTGCCTGYCCWGGGAGAGACCGGCG>70
  1>~~~~~~~~~~~~~~~~~~~~~~~~~~~~~~~~ARTGGTAATCTACTGGGACGGAACAGCTTTGAGGTGCGTGTTTGTGCCTGTCCTGGGAGAGACCGGCG>68
  1>TCTTGCTTCTCTTTTCCTATCCTGAG-----TARTGGTAATCTACTGGGACGGAACAGCTTTGAGGTGCGTGTTTGTGCCTGTCCTGGGARAGACCGGCG>95
  1>CTGCCTCTTGCTTCTCTTTTCCTATCCTGAGTARTGGTAATCTACTGGGACGGAACAGCTTTGAGGTGCGTGTTTGTGCCTGTCCTGGGAGAGACCGGCG>100
  1>TCTTGCTTCTCTTTTCCTATCCTGAG-----TARTGGTAATCTACTGGGACGGAACAGCTTTGAGGTGCGTGTTTGTGCCTGTCCTGGGAGAGACCGGCG>95
  1>CTGCCTCTTGCTTCTCTTTTCCTATCCTGAGTARTGGTAATCTACTGGGACGGAACAGCTTTGAGGTGCGTGTTTGTGCCTGTCCTGGGARAGACCGGCG>100
  1>CTTGCTTCTCTTTTCCTATCCTGRG------TARTGGTAATCTACTGGGACGGAACAGCTTTGAGGTGCGTGTTTGTGCCTGTCCTGGGAGAGACCGGCG>94
  1>CTCTTGCTTCTCTTTTCCTATCCTGRG----TARTGGTAATCTACTGGGACGGAACAGCTTTGAGGTGCGTGTTTGTGCCTGTCCTGGGAGAGACCGGCG>96
  1>CTCTTGCTTCTCTTTTCCTATCCTGAG----TARTGGTAATCTACTGGGACGGAACAGCTTTGAGGTGCGTGTTTGTGCCTGTCCTGGGAGAGACCGGCG>96

             *         *         *         *         *         *         *         *         *         *
 70>CACAGAGGAAGAGAATCTCCGCAAGAAAGGGGAGCCTCACCACGAGCTGCCCCCAGGGAGCACTAAGCGAGGTAAGCAAGCAGGACAAGAAGCGGTGGAG>169
 70>CACAGAGGAAGAGAATCTCCGCAAGAAAGGGGAGCCTCACCACGAGCTGCCCCCAGGGAGCACTAAGCGAGGTAAGCAAGCAGGACAAGAAGCGGTGGAG>169
 68>CACAGAGGAAGAGAATCTCCGCAAGAAAGGGGAGCCTCACCACGAGCTGCCCCCAGGGAGCACTAAGCGAGGTAAGCAAGCAGGACAAGAAGCGGTGGAG>167
 71>CACAGAGGAAGAGAATCTCCGCAAGAAAGGGGAGCCTCACCACGAGCTGCCCCCAGGGAGCACTAAGCGAGGTAAGCAAGCAGGACAAGAAGCGGTGGAG>170
 69>CACAGAGGAAGAGAATCTCCGCAAGAAAGGGGAGCCTCACCACGAGCTGCCCCCAGGGAGCACTAAGCGAGGTAAGCAAGCAGGACAAGAAGCGGTGGAG>168
 96>CACAGAGGAAGAGAATCTCCGCAAGAAAGGGGAGCCTCACCACGAGCTGCCCCCAGGGAGCACTAAGCGAGGTAAGCAAGCAGGACAAGAAGCGGTGGAG>195
101>CACAGAGGAAGAGAATCTCCGCAAGAAAGGGGAGCCTCACCACGAGCTGCCCCCAGGGAGCACTAAGCGAGGTAAGCAAGCAGGACAAGAAGCGGTGGAG>200
 96>CACAGAGGAAGAGAATCTCCGCAAGAAAGGGGAGCCTCACCACGAGCTGCCCCCAGGGAGCACTAAGCGAGGTAAGCAAGCAGGACAAGAAGCGGTGGAG>195
101>CACAGAGGAAGAGAATCTCCGCAAGAAAGGGGAGCCTCACCACGAGCTGCCCCCAGGGAGCACTAAGCGAGGTAAGCAAGCAGGACAAGAAGCGGTGGAG>200
 95>CACAGAGGAAGAGAATCTCCGCAAGAAAGGGGAGCCTCACCACGAGCTGCCCCCAGGGAGCACTAAGCGAGGTAAGCAAGCAGGACAAGAAGCGGTGGAG>194
 97>CACAGAGGAAGAGAATCTCCGCAAGAAAGGGGAGCCTCACCACGAGCTGCCCCCAGGGAGCACTAAGCGAGGTAAGCAAGCAGGACAAGAAGCGGTGGAG>196
 97>CACAGAGGAAGAGAATCTCCGCAAGAAAGGGGAGCCTCACCACGAGCTGCCCCCAGGGAGCACTAAGCGAGGTAAGCAAGCAGGACAAGAAGCGGTGGAG>196

             *         *         *         *         *         *         *         *
170>GAGACCAAGGGTGCAGTTATGCCTCAGATTCACTTTTATCACCTTTCCTTGCCTCTTTCCTAGCACTGCCCAACAACACCAA~~~~~>251
170>GAGACCAAGGGTGCAGTTATGCCTCAGATTCACTTTTATCACCTTTCCTTGCCTCTTTCCTAGCACTGCCCAACAACACCAA~~~~~>251
168>GAGACCAAGGGTGCAGTTATGCCTCAGATTCACTTTTATCACCTTTCCTTGCCTCTTTCCTAGCACTGCCCAACAACACCAAAASGC>254
171>GAGACCAAGGGTGCAGTTATGCCTCAGATTCACTTTTATCACCTTTCCTTGCCTCTTTCCTAGCACTGCCCAACAACACCAA~~~~~>252
169>GAGACCAAGGGTGCAGTTATGCCTCAGATTCACTTTTATCACCTTTCCTTGCCTCTTTCCTAGCACTGCCCAACAACACCAAAAGG~>254
196>GAGACCAAGGGTGCAGTTATGCCTCAGATTCACTTTTATCACCTTTCCTTGCCTCTTTCCTAGCACTGCCCAACAACACCAA~~~~~>277
201>GAGACCAAGGGTGCAGTTATGCCTCAGATTCACTTTTATCACCTTTCCTTGCCTCTTTCCTAGCACTGCCCAACAACACCAA~~~~~>282
196>GAGACCAAGGGTGCAGTTATGCCTCAGATTCACTTTTATCACCTTTCCTTGCCTCTTTCCTAGCACTGCCCAACAACACCAA~~~~~>277
201>GAGACCAAGGGTGCAGTTATGCCTCAGATTCACTTTTATCACCTTTCCTTGCCTCTTTCCTAGCACTGCCCAACAACACCAA~~~~~>282
195>GAGACCAAGGGTGCAGTTATGCCTCAGATTCACTTTTATCACCTTTCCTTGCCTCTTTCCTAGCACTGCCCAACAACACCAA~~~~~>276
197>GAGACCAAGGGTGCAGTTATGCCTCAGATTCACTTTTATCACCTTTCCTTGCCTCTTTCCTAGCACTGCCCAACAACACCAAAGAA~>282
197>GAGACCAAGGGTGCAGTTATGCCTCAGATTCACTTTTATCACCTTTCCTTGCCTCTTTCCTAGCACTGCCCAACAACACCAA~~~~~>278
```

```
Thu Jun 05, 2025 19:14 CEST
BC-PIK3CA--012.fasta from 1 to 143
Alignment to
BC-PIK3CA--011.fasta--  Matches:140; Mismatches:1; Gaps:2
BC-PIK3CA--010.fasta--  Matches:142; Mismatches:1; Gaps:0
BC-PIK3CA--009.fasta--  Matches:143; Mismatches:0; Gaps:0
BC-PIK3CA--008.fasta--  Matches:142; Mismatches:0; Gaps:1
BC-PIK3CA--007.fasta--  Matches:141; Mismatches:1; Gaps:1
BC-PIK3CA--006.fasta--  Matches:136; Mismatches:1; Gaps:3
BC-PIK3CA--005.fasta--  Matches:140; Mismatches:0; Gaps:3
BC-PIK3CA--004.fasta--  Matches:141; Mismatches:1; Gaps:1
BC-PIK3CA--003.fasta--  Matches:141; Mismatches:0; Gaps:1
BC-PIK3CA--002.fasta--  Matches:137; Mismatches:1; Gaps:2
BC-PIK3CA--001.fasta--  Matches:140; Mismatches:0; Gaps:3
```

```
            *         *         *         *         *         *         *         *         *         *
 1>~~~~~~~~~~~~~~~GATATTATTTATTTTACAGAGTAACAGACTAGCTAGAGACAATGAATTAAGGGAAAATGACAAAGAACAGCTCAAAGCAATTTC>85
 1>~~~~~~~~~~~~~~~SA-ATTATTTATTTTACAGAGTAACAGACTAGCTAGAGACAATGAATTAAGGGAAAATGACAAAGAACAGCTCAAAGCAATTTC>84
 1>AAGCTATATAA----GATATTATTTATTTTACAGAGTAACAGACTAGCTAGAGACAATGAATTAAGGGAAAATGACAAAGAACAGCTCAAAGCAATTTC>96
 1>AGCTATATAA-----GATATTATTTATTTTACAGAGTAACAGACTAGCTAGAGACAATGAATTAAGGGAAAATGACAAAGAACAGCTCAAAGCAATTTC>95
 1>AAGAAAGCTATATAAGATATTATTTATTTTACAGAGTAACAGACTAGCTAGAGACAATGAATTAAGGGAAAATGACAAAGAACAGCTCAAAGCAATTTC>100
 1>GAAAGCTATATAA--GATATTATTTATTTTACAGAGTAACAGACTAGCTAGAGACAATGAATTAAGGGAAAATGACAAAGAACAGCTCAAAGCAATTTC>98
 1>~~~~~~~~~~~~~~~~~~ATTATWTTATTTTACAGAGTAACAGACTAGCTAGAGACAATGAATTAAGGGAAAATGACAAAGAACAGCTCAAAGCAATTTC>82
 1>CTATATAA-------GATATTATTTATTTTACAGAGTAACAGACTAGCTAGAGACAATGAATTAAGGGAAAATGACAAAGAACAGCTCAAAGCAATTTC>93
 1>AAGCTATATAA----GATATTATTTATTTTACAGAGTAACAGACTAGCTAGAGACAATGAATTAAGGGAAAATGACAAAGAACAGCTCAAAGCAATTTC>96
 1>~~~~~~~~~~~~~~~~ATATTATTTATTTTACAGAGTAACAGACTAGCTAGAGACAATGAATTAAGGGAAAATGACAAAGAACAGCTCAAAGCAATTTC>84
 1>~~~~~~~~~~~~~~~~~~ATTATWTTATTTTACAGAGTAACAGACTAGCTAGAGACAATGAATTAAGGGAAAATGACAAAGAACAGCTCAAAGCAATTTC>82
 1>CTATATAA-------GATATTATTTATTTTACAGAGTAACAGACTAGCTAGAGACAATGAATTAAGGGAAAATGACAAAGAACAGCTCAAAGCAATTTC>93

                        E542     E545
             *         *         *         *         *         *
 86>TACACGAGATCCTCTCTCTGAAATCACTGMGCAGGAGAAAGATTTTCTATGGASYCMC~~~~~~~>143
 85>TACACGAGATCCTCTCTCTGAAATCACTGMGCAGGAGAAAGATTTTCTATGGAS-CMC~~~~~~~>141
 97>TACACGAGATCCTCTCTCTGAAATCACTGMGCAGGAGAAAGATTTTCTATGGASYMMC~~~~~~~>154
 96>TACACGAGATCCTCTCTCTGAAATCACTGMGCAGGAGAAAGATTTTCTATGGASYCMCRGG~~~~>156
101>TACACGAGATCCTCTCTCTGAAATCACTGMGCAGGAGAAAGATTTTCTATGGAS-CMC~~~~~~~>157
 99>TACACGAGATCCTCTCTCTGAAATCACTGMGCAGGAGAAAGATTTTCTATGGAS-CMM~~~~~~~>155
 83>TACACGAGATCCTCTCTCTGAAATCACTGMGCAGGAGAAAGATTTTCTATGGA--C-C~~~~~~~>137
 94>TACACGAGATCCTCTCTCTGAAATCACTGMGCAGGAGAAAGATTTTCTATGGA--C-C~~~~~~~>148
 97>TACACGAGATCCTCTCTCTGAAATCACTGMGCAGGAGAAAGATTTTCTATGGA-CCMCMGGGAAR>160
 85>TACACGAGATCCTCTCTCTGAAATCACTGMGCAGGAGAAAGATTTTCTATGGASYC-CCGGGWA~>147
 83>TACACGAGATCCTCTCTCTGAAATCACTGMGCAGGAGAAAGATTTTCTATGGAS-C-CCCGGGWA>145
 94>TACACGAGATCCTCTCTCTGAAATCACTGMGCAGGAGAAAGATTTTCTATGGA--C-C~~~~~~~>148
```

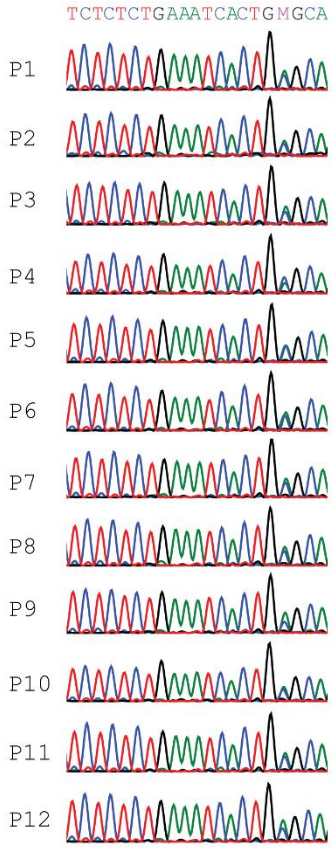

```
Mon Jun 02, 2025 15:14 CEST
BC-ESR1-012.fasta from 1 to 209
Alignment to
BC-ESR1-011.fasta--  Matches:209; Mismatches:0; Gaps:0
BC-ESR1-010.fasta--  Matches:202; Mismatches:0; Gaps:0
BC-ESR1-009.fasta--  Matches:209; Mismatches:0; Gaps:0
BC-ESR1-008.fasta--  Matches:209; Mismatches:0; Gaps:0
BC-ESR1-007.fasta--  Matches:209; Mismatches:0; Gaps:0
BC-ESR1-006.fasta--  Matches:209; Mismatches:0; Gaps:0
BC-ESR1-005.fasta--  Matches:209; Mismatches:0; Gaps:0
BC-ESR1-004.fasta--  Matches:209; Mismatches:0; Gaps:0
BC-ESR1-003.fasta--  Matches:209; Mismatches:0; Gaps:0
BC-ESR1-002.fasta--  Matches:209; Mismatches:0; Gaps:0
BC-ESR1-001.fasta--  Matches:209; Mismatches:0; Gaps:0
                                                                                                              Y537
             *         *         *         *         *         *         *         *         *         *
  1>~~~~~~~~~~AAAGTAGTCCTTTCTGTGTCTTCCCACCTACAGTAACAAAGGCATGGAGCATCTGTACAGCATGAAGTGCAAGAACGTGGTGCCCCTCTA>90
  1>CT--------AAAGTAGTCCTTTCTGTGTCTTCCCACCTACAGTAACAAAGGCATGGAGCATCTGTACAGCATGAAGTGCAAGAACGTGGTGCCCCTCTA>92
  1>~~~~~~~~~~~~~~~~~TCCTTTCTGTGTCTTCCCACCTACAGTAACAAAGGCATGGAGCATCTGTACAGCATGAAGTGCAAGAACGTGGTGCCCCTCTA>83
  1>GGTTGGCTCTAAAGTAGTCCTTTCTGTGTCTTCCCACCTACAGTAACAAAGGCATGGAGCATCTGTACAGCATGAAGTGCAAGAACGTGGTGCCCCTCTA>100
  1>T---------AAAGTAGTCCTTTCTGTGTCTTCCCACCTACAGTAACAAAGGCATGGAGCATCTGTACAGCATGAAGTGCAAGAACGTGGTGCCCCTCTA>91
  1>GCWCT-----AAAGTAGTCCTTTCTGTGTCTTCCCACCTACAGTAACAAAGGCATGGAGCATCTGTACAGCATGAAGTGCAAGAACGTGGTGCCCCTCTA>95
  1>GTTGGCTCT-AAAGTAGTCCTTTCTGTGTCTTCCCACCTACAGTAACAAAGGCATGGAGCATCTGTACAGCATGAAGTGCAAGAACGTGGTGCCCCTCTA>99
  1>TCT-------AAAGTAGTCCTTTCTGTGTCTTCCCACCTACAGTAACAAAGGCATGGAGCATCTGTACAGCATGAAGTGCAAGAACGTGGTGCCCCTCTA>93
  1>TMT-------AAAGTAGTCCTTTCTGTGTCTTCCCACCTACAGTAACAAAGGCATGGAGCATCTGTACAGCATGAAGTGCAAGAACGTGGTGCCCCTCTA>93
  1>TCT-------AAAGTAGTCCTTTCTGTGTCTTCCCACCTACAGTAACAAAGGCATGGAGCATCTGTACAGCATGAAGTGCAAGAACGTGGTGCCCCTCTA>93
  1>T---------AAAGTAGTCCTTTCTGTGTCTTCCCACCTACAGTAACAAAGGCATGGAGCATCTGTACAGCATGAAGTGCAAGAACGTGGTGCCCCTCTA>91
  1>CT--------AAAGTAGTCCTTTCTGTGTCTTCCCACCTACAGTAACAAAGGCATGGAGCATCTGTACAGCATGAAGTGCAAGAACGTGGTGCCCCTCTA>92

   D538
             *         *         *         *         *         *         *         *         *         *
 91>TGACCTGCTGCTGGAGATGCTGGACGCCCACCGCCTACATGCGCCCACTAGCCGTGGAGGGGCATCCGTGGAGGAGACGGACCAAAGCCACTTGGCCACT>190
 93>TGACCTGCTGCTGGAGATGCTGGACGCCCACCGCCTACATGCGCCCACTAGCCGTGGAGGGGCATCCGTGGAGGAGACGGACCAAAGCCACTTGGCCACT>192
 84>TGACCTGCTGCTGGAGATGCTGGACGCCCACCGCCTACATGCGCCCACTAGCCGTGGAGGGGCATCCGTGGAGGAGACGGACCAAAGCCACTTGGCCACT>183
101>TGACCTGCTGCTGGAGATGCTGGACGCCCACCGCCTACATGCGCCCACTAGCCGTGGAGGGGCATCCGTGGAGGAGACGGACCAAAGCCACTTGGCCACT>200
 92>TGACCTGCTGCTGGAGATGCTGGACGCCCACCGCCTACATGCGCCCACTAGCCGTGGAGGGGCATCCGTGGAGGAGACGGACCAAAGCCACTTGGCCACT>191
 96>TGACCTGCTGCTGGAGATGCTGGACGCCCACCGCCTACATGCGCCCACTAGCCGTGGAGGGGCATCCGTGGAGGAGACGGACCAAAGCCACTTGGCCACT>195
100>TGACCTGCTGCTGGAGATGCTGGACGCCCACCGCCTACATGCGCCCACTAGCCGTGGAGGGGCATCCGTGGAGGAGACGGACCAAAGCCACTTGGCCACT>199
 94>TGACCTGCTGCTGGAGATGCTGGACGCCCACCGCCTACATGCGCCCACTAGCCGTGGAGGGGCATCCGTGGAGGAGACGGACCAAAGCCACTTGGCCACT>193
 94>TGACCTGCTGCTGGAGATGCTGGACGCCCACCGCCTACATGCGCCCACTAGCCGTGGAGGGGCATCCGTGGAGGAGACGGACCAAAGCCACTTGGCCACT>193
 94>TGACCTGCTGCTGGAGATGCTGGACGCCCACCGCCTACATGCGCCCACTAGCCGTGGAGGGGCATCCGTGGAGGAGACGGACCAAAGCCACTTGGCCACT>193
 92>TGACCTGCTGCTGGAGATGCTGGACGCCCACCGCCTACATGCGCCCACTAGCCGTGGAGGGGCATCCGTGGAGGAGACGGACCAAAGCCACTTGGCCACT>191
 93>TGACCTGCTGCTGGAGATGCTGGACGCCCACCGCCTACATGCGCCCACTAGCCGTGGAGGGGCATCCGTGGAGGAGACGGACCAAAGCCACTTGGCCACT>192

             *         *
191>GCGGGCTCTACTTCATCGC~~~~~>209
193>GCGGGCTCTACTTCATCGC~~~~~>211
184>GCGGGCTCTACTTCATCGCAARGA>207
201>GCGGGCTCTACTTCATCGCA~~~~>220
192>GCGGGCTCTACTTCATCGC~~~~~>210
196>GCGGGCTCTACTTCATCGCAAAGC>219
200>GCGGGCTCTACTTCATCGCA~~~~>219
194>GCGGGCTCTACTTCATCGC~~~~~>212
194>GCGGGCTCTACTTCATCGC~~~~~>212
194>GCGGGCTCTACTTCATCGC~~~~~>212
192>GCGGGCTCTACTTCATCGCAATRC>215
193>GCGGGCTCTACTTCATCGCA~~~~>212
```
